# Prognostic characteristics of immune subtypes associated with acute myeloid leukemia and their identification in cell subsets based on single-cell sequencing analysis

**DOI:** 10.3389/fcell.2022.990034

**Published:** 2022-09-23

**Authors:** Jie Lu, Guowei Zheng, Ani Dong, Xinyu Chang, Xiting Cao, Mengying Liu, Xuezhong Shi, Chunmei Wang, Yongli Yang, Xiaocan Jia

**Affiliations:** ^1^ Department of Epidemiology and Biostatistics, College of Public Health, Zhengzhou University, Zhengzhou, Henan, China; ^2^ Children’s Hospital, The First Affiliated Hospital of Zhengzhou University, Zhengzhou, China

**Keywords:** acute myelogenous leukemia, single-cell RNA-seq, prognostic model, SsGSEA, tumor immune microenvironment

## Abstract

Immune genes play an important role in the development and progression of acute myeloid leukemia (AML). However, the role of immune genes in the prognosis and microenvironment of AML remains unclear. In this study, we analyzed 151 AML patients in the TCGA database for relevant immune cell infiltration. AML patients were divided into high and low immune cell infiltration clusters based on ssGSEA results. Immune-related pathways, AML pathways and glucose metabolism pathways were enriched in the high immune cell infiltration cluster. Then we screened the differential immune genes between the two immune cell infiltration clusters. Nine prognostic immune genes were finally identified in the train set by LASSO-Cox regression. We constructed a model in the train set based on the nine prognostic immune genes and validated the predictive capability in the test set. The areas under the ROC curve of the train set and the test set for ROC at 1, 3, 5 years were 0.807, 0.813, 0.815, and 0.731, 0.745, 0.830, respectively. The areas under ROC curve of external validation set in 1, 3, and 5 years were 0.564, 0.619, and 0.614, respectively. People with high risk scores accompanied by high TMB had been detected with the worst prognosis. Single-cell sequencing analysis revealed the expression of prognostic genes in AML cell subsets and pseudo-time analysis described the differentiation trajectory of cell subsets. In conclusion, our results reveal the characteristics of immune microenvironment and cell subsets of AML, while it still needs to be confirmed in larger samples studies. The prognosis model constructed with nine key immune genes can provide a new method to assess the prognosis of AML patients.

## Introduction

Acute myeloid leukemia (AML) is a highly heterogeneous hematological malignancy characterized by uncontrolled proliferation and differentiation of hematopoietic progenitor cells/stem cells in bone marrow, blood, and other hematopoietic organs (Medinger and Passweg, 2017; Short et al., 2018). Cancer statistics 2019 showed that the 5-years relative survival rate for AML was 66.4% in children and 64.2% in adolescents from 2008 to 2014 (Siegel et al., 2019). The median overall survival (OS) after 5 years in adults (18–60 years) with AML was approximately 40% (Schlenk and Döhner, 2013). Current studies have shown that prognosis of AML is closely related to white blood cell count and cytogenetic abnormalities (Kalaiyarasi et al., 2019; Tallman et al., 2019). The main treatment strategies for AML are intensive induction chemotherapy and post-remission therapy. Although most AML patients initially achieve significant remission with chemotherapy. Complete elimination of AML cells remains rare, with no substantial improvement in patient survival (Döhner et al., 2010; Hosono, 2019). As the process of translating the relevant genomic landscape knowledge of AML into clinical treatment has only just begun, the identification of new potential biomarkers will contribute to the diagnosis and prognosis of AML patients.

AML has long been considered an immunoreactive malignancy and multiple mechanisms are implicated in AML’s immune evasion (Passweg et al., 2019). AML immune escape is caused by both intrinsic and extrinsic immunosuppressive mechanisms (Teague and Kline, 2013; Mittal et al., 2014). Intrinsic immunosuppressive effects include upregulation of anti-apoptotic mechanisms, regulation of immunomodulatory checkpoints and loss of tumor antigen expression. Extrinsic mechanisms include the accumulation of regulatory cells such as regulatory T-cells (Tregs) and secretion of immunosuppressive cytokines (Austin et al., 2016). The production of immune escape depends on the tumor microenvironment (TME). TME is a dynamic system composed of extracellular matrix, stromal cells and immune cells (Ayala et al., 2009; Roma-Rodrigues et al., 2019). Similar to most tumors, functional interactions between leukemic cells and the bone marrow immune microenvironment constitute a unique hallmark of AML (Isidori et al., 2021). Although the prognosis of AML patients currently mainly depends on cellular and molecular genetic characteristics, the TME also plays an extremely important role in the progression and treatment of AML (Yehudai-Resheff et al., 2019). It was reported that the leukemia TME inhibits the growth of normal hematopoietic cells while promoting and maintaining the proliferation and long-term viability of leukemia cells (Basak et al., 2010). Immune response in the tumor microenvironment is a significant factor in the invasion and progression of various tumors, among which immune cell types, cytokines and immune genes have been widely studied as prognostic markers in many tumors such as lung cancer, ovarian cancer and colorectal cancer (Frey et al., 2017; Lee et al., 2017). In addition, the discovery of numerous immune checkpoints also offers a broader therapeutic prospect for AML and other malignancies (Zhang et al., 2009). Therefore, identifying the characteristics of TME in AML is crucial for designing personalized immune therapy for AML patients.

Single-cell sequencing (scRNA-seq) is a common technique to explore the heterogeneity and diversity of tumor cells. It can describe the functional state of a cell by detecting the transcription level of a single cell (Petti et al., 2019). In AML, enhanced T-cell-mediated clearance of AML is an attractive therapeutic strategy, but immunotherapy trials have been less successful than in other cancers (Lichtenegger et al., 2017). ScRNA-seq provides a powerful means to characterize malignant and stromal cell populations in tumors, which may address questions related to dryness, developmental hierarchies, and interactions between malignant and immune cells (Giustacchini et al., 2017). Therefore, scRNA-seq can further explore the composition and functional status of various immune cell subsets in the AML bone marrow microenvironment (Miles et al., 2020). It could lead to exciting breakthroughs in cancer genomics in the future.

In this study, two immune cell infiltration clusters were identified based on The Cancer Genome Atlas (TCGA) database. We compared the characteristics of TME and pathway enrichment differences between the two clusters. The prognostic model was constructed using differential immune genes to accurately predict the prognosis of AML patients. ScRNA-seq was used to study the expression characteristics and differentiation trajectory of prognostic immune genes in AML cell subsets. Our comprehensive analysis of AML populations with different immune statuses may provide a new reference for the characteristics, treatment and prognosis of AML.

## Materials and methods

### Acquisition and pre-processing of gene and clinical data

RNA sequencing data of 151 AML patients’ samples and the clinical data of 200 AML patients’ samples were downloaded from TCGA database (https://www.cancer.gov/about-nci/organization/ccg/research/structural-genomics/tcga), 142 samples with both clinical information and sequencing data. In addition, transcriptome and clinical information from 422 HGU-133A AML patients in the cohort GSE37642 (https://www.ncbi.nlm.nih.gov/geo/query/acc.cgi) were downloaded from the Gene Expression Omnibus (GEO) database for external validation. Then, we downloaded the Genome Reference Consortium Human Build 38 (GRCh38) (https://www.gencodegenes.org/human/) gene annotation file and performed gene annotation for all gene probes. Single-cell sequencing data was downloaded from chip GSE126068 (https://www.ncbi.nlm.nih.gov/geo/query/acc.cgi?acc=GSE126068) which included 5 patients. The original file included 26,454 genes and 813 cells, with 400 cells detected at diagnosis and 413 cells detected at relapse. Normal blood samples were collected from the Genotype-Tissue Expression (GTEx) database (https://xenabrowser.net/datapages/?cohort=GTEX&removeHub=https%3A%2F%2Fxena.treehouse.gi.ucsc.edu%3A443). The RNA sequencing data were transcribed fragments per kilobase per million mapped reads (FPKM) normalized. We used the combat function in the “sva” package of R language to remove the batch effect of high-throughput data to eliminate the data differences caused by different platforms.

### Single sample gene set enrichment analysis algorithm and clustering of acute myeloid leukemia patients

Single sample gene set enrichment analysis (ssGSEA), an extension of Gene Set Enrichment Analysis (GSEA), calculates separate enrichment scores for each sample and gene set (Subramanian et al., 2005). In our study, ssGSEA algorithm calculated a scoring of immune cell types and immune pathways against innate and adaptive immune for each sample. Next, we used “hclust” clustering method to cluster all AML patients according to ssGSEA scores and divide them into clusters with high immune cell infiltration and low immune cell infiltration. The t-distributed stochastic neighbor embedding (t-SNE) algorithm was used to verify and visualize the clustering results.

### Comparison of clinical features and immune-related characteristics between the two cluster patients

The ESTIMATE (Estimation of Stromal and Immune cells in Malignant Tumor tissues using Expression data) (version 2.15.3) algorithm from the website (https://sourceforge.net/projects/estimateproject/) was used to calculate the estimated score, immune score and stromal score (Yoshihara et al., 2013). We used the Wilcoxon test to assess the difference between the two groups for the stromal score, immune score, and ESTIMATE score. The R packages “ggpubr” and “pheatmap” were applied to visualize our results. Next, the CIBERSORT (Cell type Identification by Estimating Relative Subpopulations of RNA Transcription) method was applied to assess the proportion of 22 immune cell subtypes in AML patient samples. Wilcoxon test was also used to evaluate the differences in the degree of infiltration of 22 immune cells between the two clusters.

### Human leukocyte antigen, genome set enrichment analysis, and clinical factors differentials analysis between the two cluster patients

A total of 24 HLAs (human leukocyte antigen), including HLA-E, HLA-DPB2, HLA-C, HLA-J, HLA-DQB1, HLA-DQB2, HLA-DQA2, HLA-DQA1, HLA-A, HLA-DMA, HLA-DOB, HLA-DRB1, HLA-H, HLA-B, HLA-DRB5, HLA-DOA, HLA-DPB1, HLA-DRA, HLA-DRB6, HLA-L, HLA-F, HLA-G, HLA-DMB, and HLA-DPA1 were acquired from a previous study (Gonzalez-Galarza et al., 2013; Mack et al., 2013). Meanwhile, we downloaded the mutation data and tumor mutation burden (TMB) data from TCGA database and further extracted them using “perl” (http://www.perl.org/) language. Similarly, we used the Wilcoxon test to assess whether HLAs and clinically relevant factors differed between the two clusters. GSEA was also performed between two clusters to find enriched biological pathways. Kyoto encyclopedia of genes and genomes (KEGG) gene sets (c2.cp.kegg.v7.4.symbols.gmt) and phenotype tag expression files were loaded into the GSEA software and run 1,000 times to demonstrate the function consistently. The screening criteria were nominal (NOM) *p*-value < 0.05 and |normalized enrichment score (NES)| > 1.

### Construction and validation of prognostic modeling by immune-related genes

The “limma” package was used to identify the different genes between two different immune clusters in the R language. FDR <0.05 and | logFC (fold change) | > 2 were used as thresholds. 1793 immune-associated genes were downloaded from the ImmPort database (https://www.immport.org/home) and further intersected with the differential genes to obtain immune-associated differential genes. We divided the AML patients into the train set and test set according to the ratio of 7:3. Next, Cox regression analysis and Least absolute shrinkage and selection operator (LASSO) regression were used to screen for immune genes associated with AML prognosis in the train set. Patients in the train, test and validation sets were assigned to the high-risk and low-risk groups based on their median risk score. The calculating formula is:

$$risk\;score=\overset{n}{\underset{i=1}{\sum }}coef_{i}*x_{i}$$

where $co\mathrm{e}f_{i}$ means the coefficients, $x_{i}$ is the FPKM value of each gene associated with the prognosis of AML patients. We constructed a prognostic model by immune-related genes and tested the survival scores, risk status and predictive power of this model for survival in AML patients by the Receiver Operating Characteristic (ROC) curve among the train set, the test set and external set. Finally, we integrated the GTEx database and TCGA data of leukemia patients, and then compared the expression levels of all the prognostic immune genes related to leukemia in normal people and leukemia patients.

### Constructing a predictive nomogram

Nomogram is widely used to predict the prognosis of cancer (Iasonos et al., 2008). All independent prognostic factors, including age, race, bone marrow blast cell percent value, hemoglobin value, monocyte percent value, leukocyte value, FAB stage and risk score were identified by univariate Cox regression and multivariate Cox regression analysis to build a nomogram to investigate the probability of 1 year, 3 years, and 5 years overall survival (OS) of AML. Finally, calibration curves were performed to determine the predictive power of the nomogram for patient survival.

### Discovery of co-expressed transcription factors and construction of PPI networks

318 transcription factors were downloaded from the Cistrome database (http://www.cistrome.org/). Pearson correlation analysis was used to find transcription factors associated with prognosis-related immune genes. We next mapped the PPI (protein-protein interaction) network by string website (https://www.string-db.org/) on the immune prognostic genes and transcription factors with co-expression relationships. The screening criteria were *R* ≥ 0.4 and *p* < 0.001.

### Differences in immune checkpoint, tumor mutation characteristics, tumor mutation burden and immunotherapy response between different prognostic risk groups

Differences in the 5 common immune checkpoints and mutation frequency between high and low risk groups were further compared by the Wilcoxon test. The five common immune checkpoints are programmed death 1 (PD-1) (Sharpe and Pauken, 2018) and its ligand 1 (PD-L1) (Daassi et al., 2020), cytotoxic T lymphocyte antigen 4 (CTLA-4) (Agdashian et al., 2019), mucin domain-containing molecule-3 (TIM-3) (Wolf et al., 2020) and lymphocyte-activation gene 3 (LAG3). Spearman correlation method was further applied to explore the correlation between risk scores and immune checkpoints. Since TMB has been identified as a biomarker for several cancer types in response to immune checkpoints (Merino et al., 2020), we further analyzed differences in TMB between high and low risk groups. Besides, to further explore the relationship between TMB and survival of AML, AML patients in the TCGA database were divided into high TMB group and low TMB group according to the median value of TMB. Kaplan-Meier survival curve was used to determine whether there was the difference in survival between high and low TMB groups. AML patients in the TCGA database were divided into four groups: high risk with high TMB group, high risk with low TMB group, low-risk with high-TMB group, and low-risk with low-TMB group based on the median risk score and median TMB. Similarly, Kaplan-Meier survival curve was used to determine whether there was difference in survival among four groups. Tumor Immune Dysfunction and Exclusion (TIDE) algorithms were widely used to predict response to ICI therapy (anti-PD-1 and CTLA-4 therapy). ICI responses and measurements were assessed in the high-risk and low-risk groups using the TIDE algorithm (Jiang et al., 2018).

### Single-cell sequencing analysis

We first integrated the expression levels of the genes in the diagnosis and relapse parts into two matrixes, respectively. Then two matrixes were transformed into “Seurat” objects and carried out quality control and standardization. The “PercentageFeatureSet” function was used to calculate the percentage of mitochondrial genes. Due to the loss of cytoplasmic RNA and leakage of mitochondria from the damaged membrane when the cell is destroyed. So, we removed more than 5% of the cells with mitochondrial genes and fewer than 50 genes. The “FindVariableFeatures” function was used to find the first 1,500 or 5,000 highly variable genes. To preserve the information of the original variables as much as possible, we used principal component analysis (PCA) to reduce the dimensionality of the data by the information of 1,500 or 5,000 highly variable genes. We included statistically significant principal components for the next subsets of cell annotation. Next, the function “FindAllMarkers” was used to identify overexpressed genomes and the “SingleR” package was used to annotate cell subsets.

### Pseudotime analysis

To study the developmental trajectory of various cells in AML during tumor development and progression, monocle (version 2.14.0, used for pseudo-time analysis) was used to analyze the gene expression matrix with Seurat annotation (Trapnell et al., 2014). In the course of a cell’s life, many cell states are not completely synchronized. Some cells are at the beginning of a particular process, while others are already in the state of completion of that process, which is called “asynchronous”. By ordering cells according to this process to form a trajectory, the process changes associated with the trajectory can be tracked as “pseudotime”. We arranged the cells in the pseudo-time analysis along the track and made heatmaps based on the prognostic immune genes. Finally, the expression of prognostic genes in different cells was described to explore their possible roles.

### Statistical analysis

All statistical analyses were conducted using the R software version 4.1.0 (http://www.R-project.org). Unless otherwise mentioned, *p* < 0.05 was regarded as statistically significant.

## Results

### Characteristics of participants in this study

**FIGURE 1:**
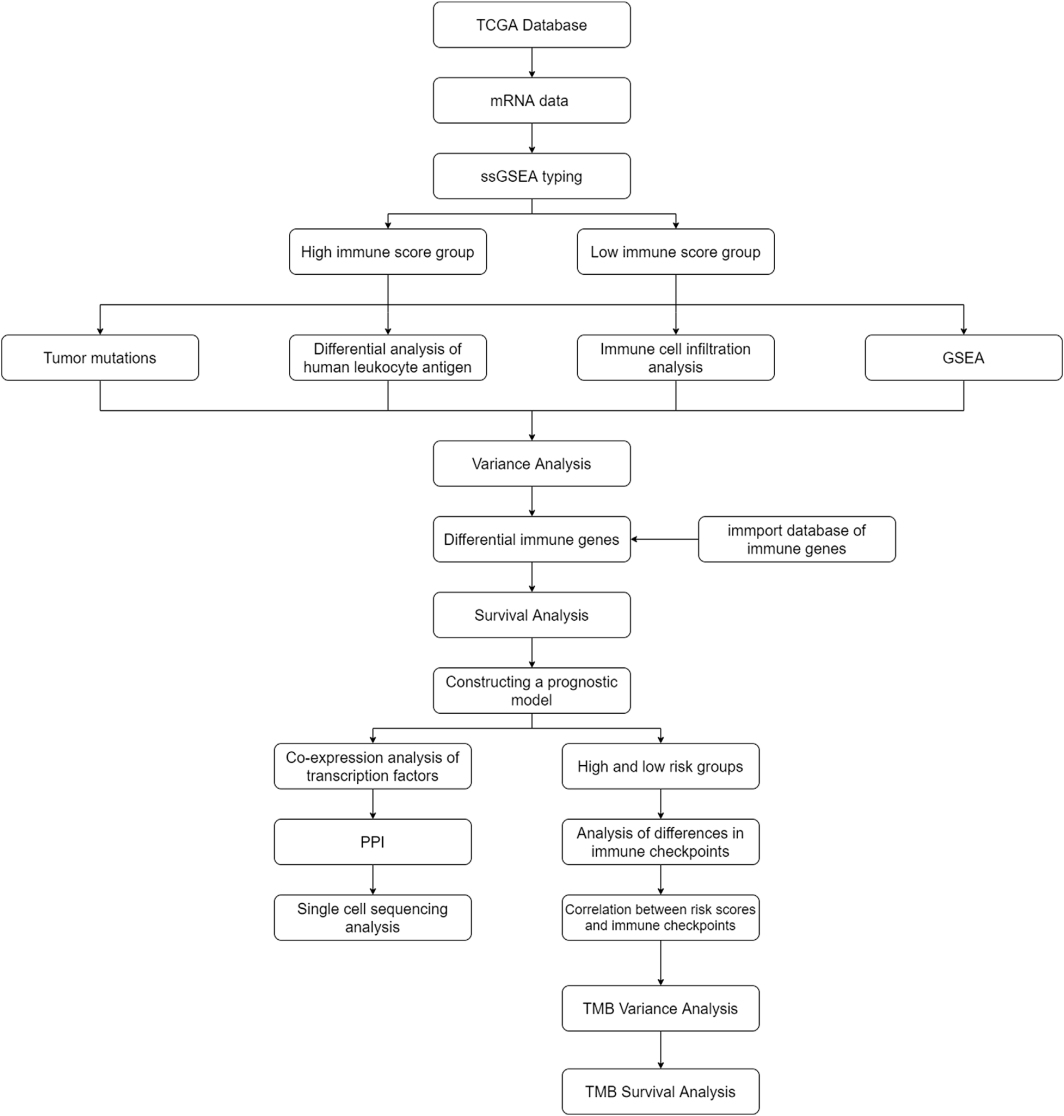
Flow chart.

Table 1 presents the baseline characteristics of AML patients in the TCGA database. Figure 1 illustrates the flow chart of this study. Baseline characteristics of the train set and test set populations are shown in Supplementary Tables S1, S2. Table 2 presents the baseline characteristics of AML patients in the GEO database.

**TABLE 1:** Baseline characteristics for 200 patients with AML in the TCGA database.

Characteristics	Cases (%)
Gender	
Female	91 (45.5)
Male	109 (54.5)
Age (year)	
10∼	1 (0.5)
20∼	16 (8.0)
30∼	21 (10.5)
40∼	26 (13.0)
50∼	44 (22.0)
60∼	54 (27.0)
70∼	32 (16.0)
80∼	6 (3.0)
Race	
Asian	2 (1.0)
Black or African American	15 (7.5)
Not reported	2 (1.0)
White	181 (90.5)
FAB Category	
M0 Undifferentiated	19 (9.5)
M1	44 (12.5)
M2	44 (12.5)
M3	21 (10.5)
M4	42 (21.0)
M5	22 (11.0)
M6	3 (1.5)
M7	3 (1.5)
Not classified	2 (1.0)
Ethnicity	
Hispanic or Latino	3 (1.5)
Not Hispanic or Latino	194 (97.0)
Not reported	3 (1.5)

**TABLE 2:** Baseline characteristics for 422 patients with AML in the GEO database (*n* = 422).

Characteristics	Cases (%)
Age (year)	
10∼	2 (0.5%)
20∼	27 (6.4%)
30∼	48 (11.4%)
40∼	69 (16.4%)
50∼	82 (19.4%)
60∼	128 (30.3%)
70∼	61 (14.5%)
80∼	5 (1.2%)
FAB Category	
M0	14 (3.3%)
M1	84 (19.9%)
M2	117 (27.7%)
M3	19 (4.5%)
M4	104 (24.6%)
M5	47 (11.1%)
M6	15 (3.6%)
M7	2 (0.5%)
NA	20 (4.7%)
Survival state	
alive	109 (25.8%)
dead	308 (73.0%)
NA	5 (1.2%)

### Single sample gene set enrichment analysis algorithm and clustering of acute myeloid leukemia patients

**FIGURE 2:**
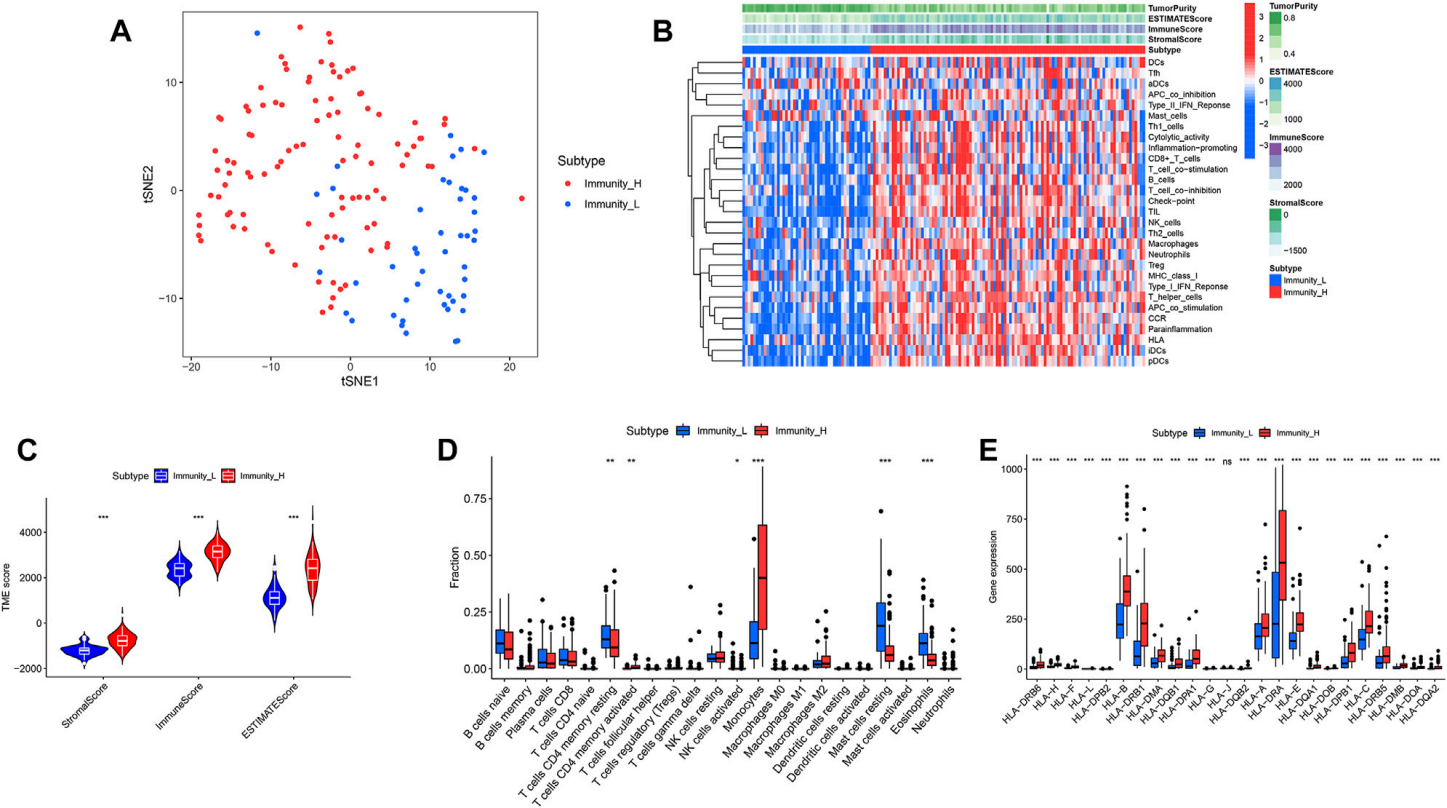
“hclust” cluster method and immune-related functions. **(A)** t-SNE was used to test the clustering results of the high and low immune groups. **(B)** Heatmaps of immune-related pathways in the high and low immune groups. **(C)** Stromal score, immune score and total score in the high and low immune groups. **(D)** Comparison of 22 kinds of immune cells in the high and low immune groups. **(E)** Comparison of human leukocyte antigen differences between high and low immune groups.

39740 RNAs from 151 AML patients were extracted and integrated into a matrix through the perl language. The ssGSEA method was applied to assess the richness levels of immune cells and immune pathways in 151 AML patients. AML patients were classified into two categories based on the results of immune infiltration, which include the high immune cell infiltration cluster (*n* = 103) and low immune cell infiltration cluster (*n* = 48) by the “hclust” clustering method. The t-SNE method was further used to visualize and validate the clustering results (Figure 2A).

### Immune cell infiltration differential analysis between the two clusters

We calculated and compared the stromal score, immune score, estimate score and tumor purity of the two clusters according to the ESTIMATE algorithm. It showed that the stromal score, immune score and estimate score of high immune cell infiltration cluster were higher than those of low immune cell infiltration cluster, while tumor purity was the opposite (Figure 2B). The violin plot showed that the stromal score, immune score and estimate score were all higher in the high immune cell infiltration cluster than in the low (Figure 2C) (*p* < 0.05). Among the contents of 22 immune cell, we found only 6 of them, including CD4 resting memory T-cells, activated CD4 memory T-cells, NK cells activated, Monocytes, Mast cells resting and Eosinophils, appearing differentially between the high and low immune cell infiltration clusters. The CD4 resting memory T-cells, NK cells activated, Mast cells resting and Eosinophils contents were higher in the low immune cell infiltration cluster than in the high immune cell infiltration cluster, while the activated CD4 memory T-cells and Monocytes contents were opposite (Figure 2D) (*p* < 0.05).

### Human leukocyte antigen, genome set enrichment analysis and clinical factors differentials analysis between the two clusters

**FIGURE 3:**
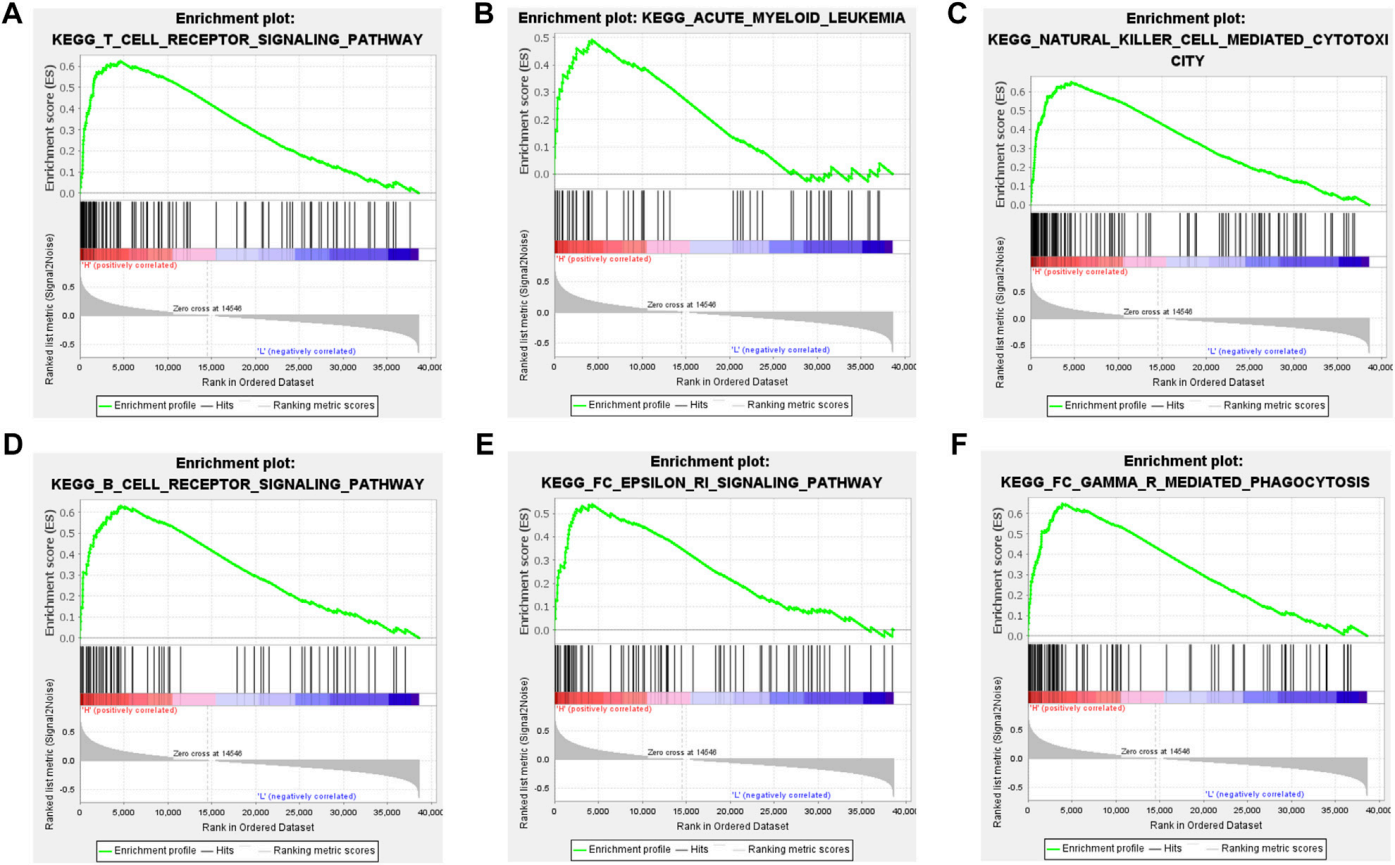
GSEA between the high and low immune groups. **(A)** T cell receptor signaling pathway. **(B)** Acute myeloid leukemia pathway. **(C)** Natural killer cell mediated cytotoxicity pathway. **(D)** B cell receptor signaling pathway. **(E)** FC epsilon ri signaling pathway. **(F)** FC gamma r mediated phagocytosis pathway.

We first compared the differences in clinical factors between high and low immune cell infiltration clusters, including monocyte percent value, age, hemoglobin value, bone marrow blast cell percent value and leukocyte value. We found that the age and monocyte percent value of the high immune cell infiltration cluster were higher than that of the low immune cell infiltration cluster, while bone marrow blast cell percent value and leukocyte value were opposite (Supplementary Figures S1A–E) (*p* < 0.05). Next, the results of the boxplot showed that the expression level of all 23 HLAs excluding HLA-J was higher in the high immune cell infiltration cluster than in the low immune cell infiltration cluster (Figure 2E) (*p* < 0.05). GSEA has shown that many immune-related pathways, such as Natural killer cell-mediated cytotoxicity, T-cell receptor signaling pathway, B cell receptor signaling pathway and Fc epsilon RI signaling pathway were enriched in the high immune cell infiltration cluster. Besides, the Insulin signaling pathway, Acute myeloid Leukemia Pathway, Regulation of actin cytoskeleton pathway and other biological pathways were also enriched in the high immune cell infiltration cluster (Figures 3A–F) (Nom *p*-value < 0.05).

### Construction and validation of prognostic modeling by immune-related genes

**FIGURE 4:**
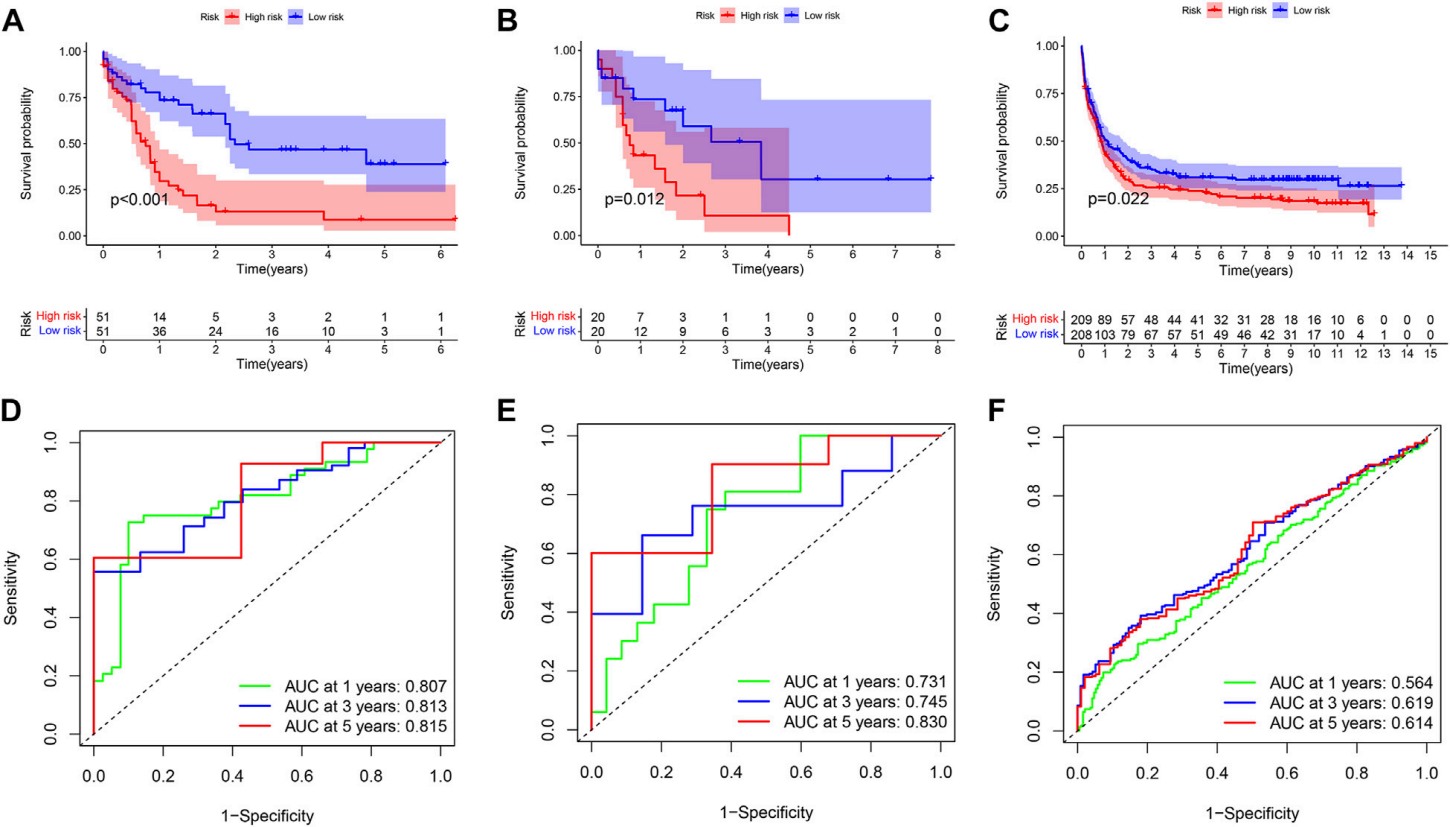
Univariate Cox regression analysis and prognosis curve. **(A)** KM curve of train set. **(B)** KM curve of test set. **(C)** KM curve of GEO external validation set. **(D)** ROC curves of model for predicting the 1/3/5-years survival in the train set. **(E)** ROC curves of model for predicting the 1/3/5-years survival in the test set. **(F)** ROC curves of model for predicting the 1/3/5-years survival in the GEO external validation set.

**FIGURE 5:**
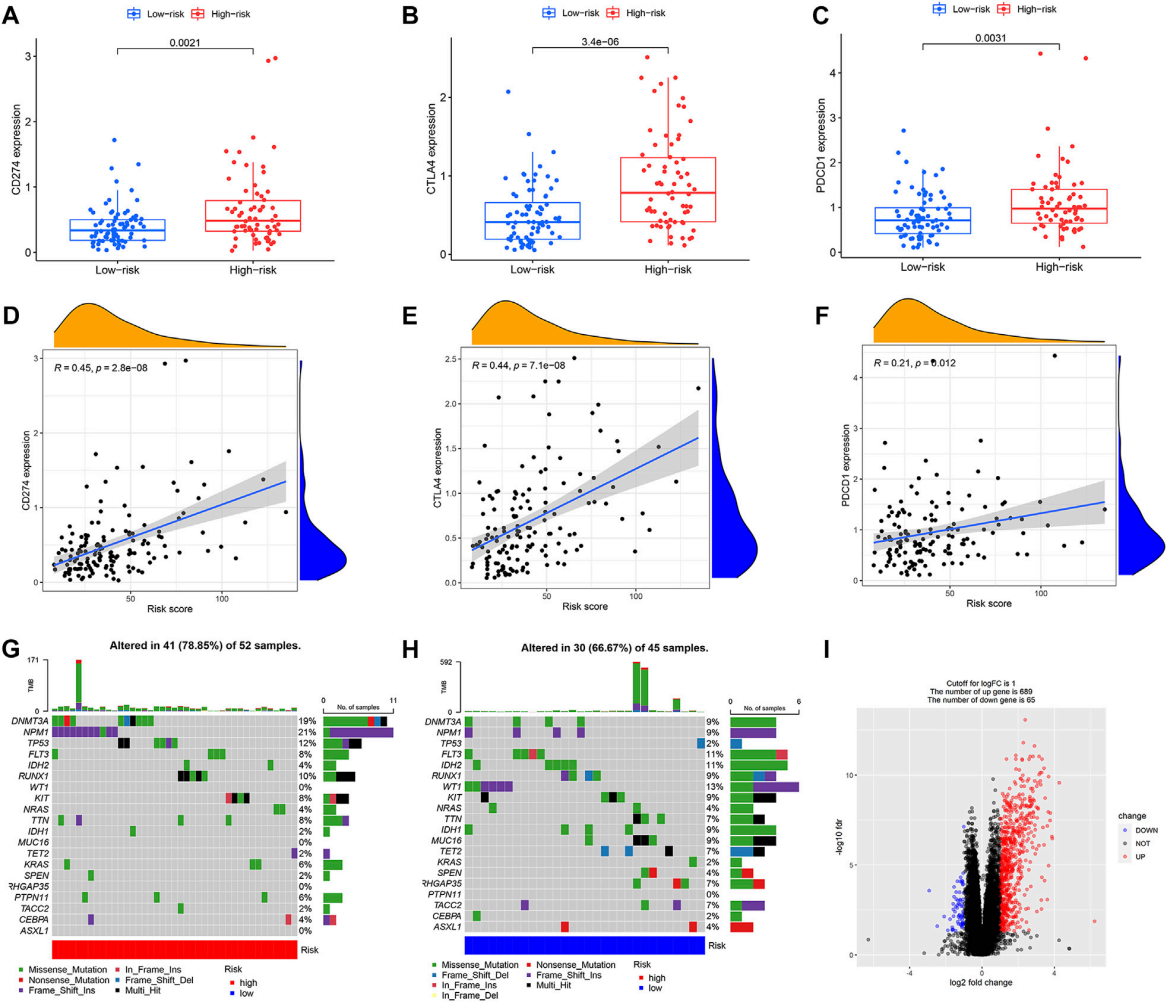
Characteristics of mutations and immune checkpoints between high and low risk groups. **(A)** Expression of CD274 between high and low risk groups. **(B)** Expression of CTLA4 between high and low risk groups. **(C)** Expression of PDCD1 between high and low risk groups. **(D)** Correlation between CD274 and risk score. **(E)** Correlation between CTLA4 and risk score. **(F)** Correlation between PDCD1 and risk score. **(G)** Tumor mutations in the high-risk group. **(H)** Tumor mutations in the low-risk group. **(I)** Volcano map of differential gene screening.

689 genes upregulated in the high immune group and 65 genes downregulated in the high immune cell infiltration cluster were screened by differential analysis (Figure 5). We next took intersections of the 754 differential genes with 1793 immune-related genes. 183 intersecting genes were obtained using Venn analysis (Supplementary Figure S2C). We integrated 183 gene expression matrixes from 151 AML patients with clinical information from 200 AML patients. 142 AML patients with complete clinical data were eventually obtained. 39 genes were significantly associated with overall survival in AML patients using univariate Cox regression (Supplementary Figure S2I) (*p* < 0.05). Nine genes including CD74, PLXNB1, THBS1, PTK2, UNC93B1, PPBP, CXCL12, GZMB, and IFI30 were finally identified by LASSO regression analysis to construct the prognostic model. Risk score = (0.00239 * CD74) + (−0.13147 * PLXNB1) + (0.0166 * THBS1) + (−0.11492 * PTK2) + (0.01322 * UNC93B1) + (0.00479 * PPBP) + (−0.01512 * CXCL12) + (0.03528 * GZMB) + (−0.048 * IFI30). The coefficients result was also shown in Table 3. Then, we divided all AML patients into the train set and the test set according to the ratio of 7:3. AML patients in the train set were divided into high-risk and low-risk groups based on the median risk score. Kaplan-Meier (KM) curve showed that the survival rate of patients in the low-risk group was significantly higher than that in the high-risk group (Figure 4A) (*p* < 0.01). Scatter plot results of risk score and survival status of AML patients showed that the mortality and risk coefficient was lower in the low-risk group than in the high-risk group (Supplementary Figures S1H,I). The AUC of the risk score predicted OS at 1-, 3- and 5- year were 0.807, 0.813, and 0.815, respectively, which means our signature has a good capacity in predicting OS (Figure 4D). The predictive power of our model was also verified in the test set. The KM curve showed that patients in the low-risk group had higher survival rates than those in the high-risk group (Figure 4B) (*p* = 0.012), while lower mortality and risk factors than those in the high-risk group (Supplementary Figures S1J,K). The AUC of the risk score predicted OS at 1-, 3- and 5- year were 0.731, 0.745, and 0.830, respectively (Figure 4E). The calibration curves of the train set and test set were shown in Supplementary Figures S1F,G, respectively. Then, we verify the predictive ability of our model in the GEO external validation set. KM curve results showed that there was a statistical difference in OS between high-risk and low-risk groups (*p* = 0.022) (Figure 4C). The areas under ROC curve in 1, 3 and 5 years were 0.564, 0.619, and 0.614, respectively (Figure 4F). Finally, we compared the expression of 9 immune genes related to the prognosis of leukemia in normal blood samples and leukemia patients. The expression levels of CXCL12, THBS1, PPBP, GZMB, CD74, and UNC93B1 were higher in the cancer group than in the normal group. The expression levels of PLXNB1, PTK2, and IFI30 in the cancer group were lower than those in the normal group (Supplementary Figures S5A,B).

**TABLE 3:** Coefficient of 9 immune-related prognostic genes.

Gene	Coefficient	p-value
CD74	0.00239	<0.05
PLXNB1	−0.13147	<0.05
THBS1	0.0166	<0.05
PTK2	−0.11492	<0.05
UNC93B1	0.01322	<0.05
PPBP	0.00479	<0.05
CXCL12	−0.01512	<0.05
GZMB	0.03528	<0.05
IFI30	−0.048	<0.05

### Constructing a predictive nomogram

**FIGURE 6:**
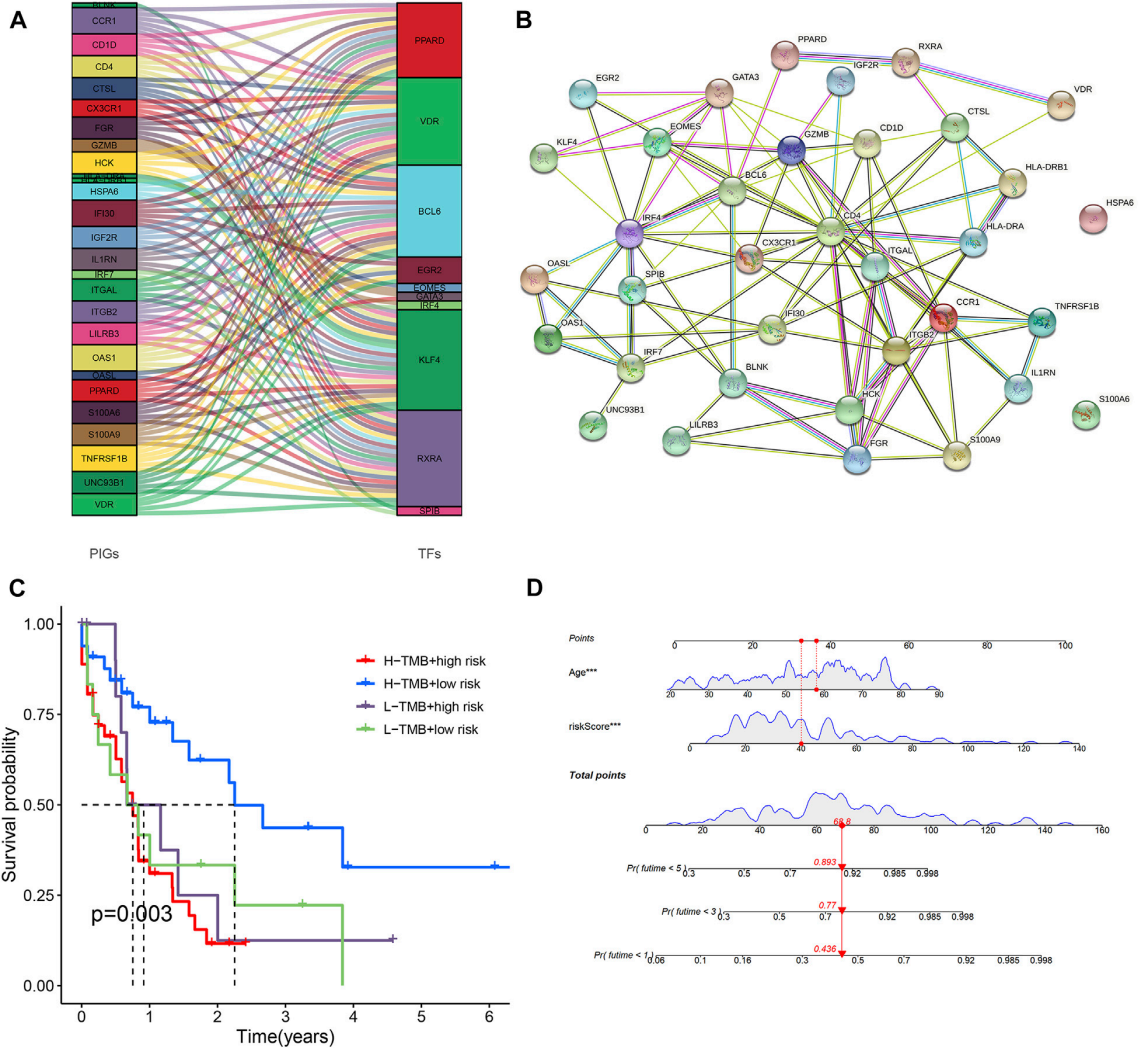
Predictive nomogram and PPI network. **(A)** Analysis of co-expressed of transcription factors and immune genes. **(B)** PPI networks of transcription factors and immune genes. **(C)** Comparison of survival rates for different TMB and different risk levels. **(D)** All clinical indicators were included in the nomogram.

Nomogram was designed based on all AML patients to predict the survival probability of patients at 1 year, 3 years, and 5 years. Multivariate Cox regression results showed that age and risk score were prognostic factors for AML patients. Risk score and age were included as variables. Age and risk scores were found to be significantly associated with AML prognosis (Figure 6) (Supplementary Figures S5C,D) (*p* < 0.01).

### Discovery of co-expressed transcription factors and construction of PPI networks

318 transcription factors and 754 genes were used for co-expression analysis. 27 mRNAs and 10 transcription factors were found to be co-expressed (*R* = 0.4, *p* < 0.001). Figure 6A showed the relationship between transcription factors and genes. We used the STRING online database to map the PPI network using the 35 hub nodes, including 27 mRNAs and 10 transcription factors (Figure 6B) (*R* = 0.4).

### Differences in immune checkpoint, tumor mutation characteristics, tumor mutation burden and immunotherapy response between different prognostic risk groups

We further compared differences in immune checkpoints, tumor mutation characteristics, and TMB between the high-risk and low-risk groups. The results of the boxplot showed that the expression levels of CD274, CTLA4, HAVCR2, LAG3, and PDCD1 in the high immune cell infiltration cluster were higher than those in the low immune cell infiltration cluster (Figures 5A−C) (Supplementary Figures S2A,B) (*p* < 0.05). The results of the scatter plot showed a positive correlation between the expression of CD274, CTLA4, HAVCR2, LAG3, and PDCD1 and the risk score (Figures 5D−F) (Supplementary Figures S2D,E) (*p* < 0.05). There was no difference in tumor mutation load between the high-risk and low-risk groups (Supplementary Figure S2H) (*p* = 0.37). Survival analysis showed that there was no difference in prognosis between the high and low tumor mutation load group (Supplementary Figure S2G) (*p* = 0.375). More importantly, TMB survival curves combined with risk scores showed that patients in the high-mutation and high-risk groups had the worst survival outcomes (Figure 6C) (*p* < 0.01). The frequency of gene mutations was higher in the high-risk group (78.85%) than in the low-risk group (66.67%). We found the highest mutation frequency in NPM1 in the high-risk group and the highest mutation frequency in WT1 in the low-risk group (Figures 5G,H). The results of immunotherapy showed that MSI and Exclusion were higher in the low-risk group, while Dysfunction and TIDE were higher in the high-risk group (Supplementary Figures S6A−D).

### Single-cell sequencing analysis

**FIGURE 7:**
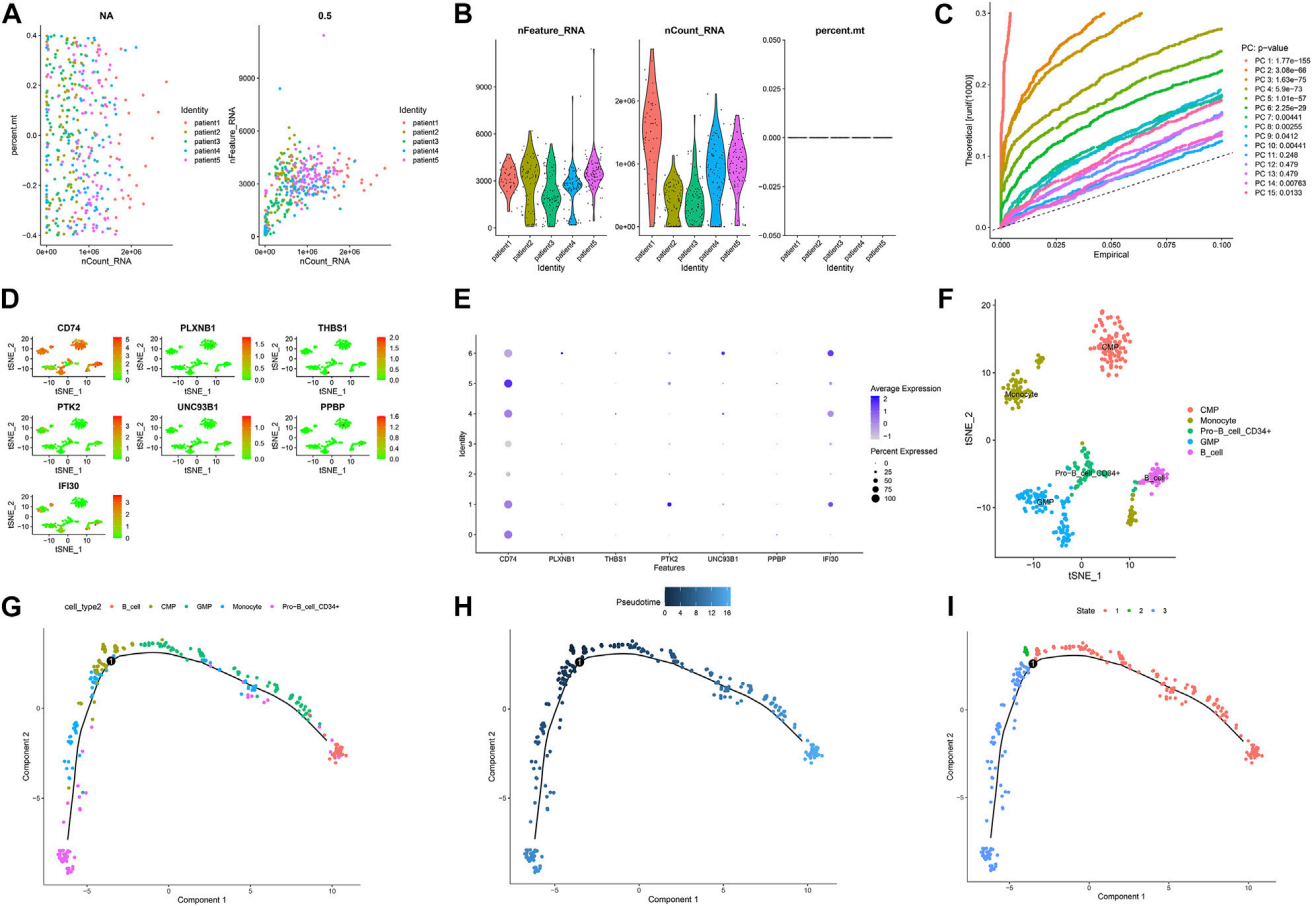
Single-cell sequencing results and pseudotime differentiation in AML patients at diagnosis. **(A)** RNA count charts of 5 patients. **(B)** Sequencing depth, gene count, and mitochondrial content of 5 patients. **(C)** PCA dimension reduction was performed on characteristic genes. **(D–E)** Expression of 7 prognostic genes in cell subsets. **(F)** Annotation results of cell subsets. **(G)** Sequence of different cell clusters along pseudo-time. Infer cell sequence from expression of the most dispersed genes in the cell cluster. Each point corresponds to a cell, and each color represents a state. **(H)** The darker the blue, the earlier the differentiation, and the lighter the blue, the later the differentiation. **(I)** Each color represents a different state.

**FIGURE 8:**
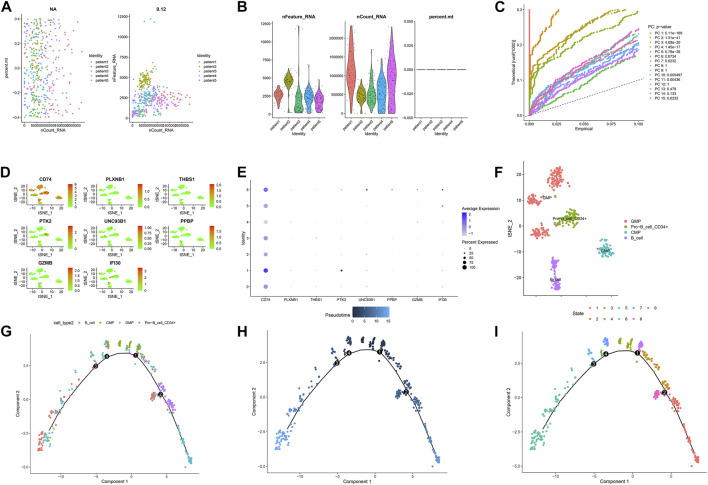
Single-cell sequencing results and pseudotime differentiation in AML patients at relapse. **(A)** RNA count charts of 5 patients. **(B)** Sequencing depth, gene count, and mitochondrial content of 5 patients. **(C)** PCA dimension reduction was performed on characteristic genes. **(D–E)** Expression of 7 prognostic genes in cell subsets. **(F)** Annotation results of cell subsets. **(G)** Sequence of different cell clusters along pseudo-time. Infer cell sequence from expression of the most dispersed genes in the cell cluster. Each point corresponds to a cell, and each color represents a state. **(H)** The darker the blue, the earlier the differentiation, and the lighter the blue, the later the differentiation. **(I)** Each color represents a different state.

Figures 7A,B and Figures 8A,B showed the Chip quality control in diagnosed and relapsed populations, respectively. We separately performed the “ScaleData” function to scale all the genes extracted from the scRNA-seq dataset GSE126068. Expression of immune-related prognostic genes in cell subsets of the diagnosed and relapsed population was shown in Figures 7D,E and Figures 8D,E. PCA selected the first 15 principal components to screen out possibly rarer cell subsets (Figures 7C, 8C). The 400 cells at diagnosis were divided into seven cell subsets. Seven cell subgroups were annotated and divided into five cell types, including common myeloid progenitor (CMP), Granulocyte-Monocyte progenitor (GMP), B cell, Pro-B_CELL_CD34 + and Monocyte (Figure 7F). The 413 cells at relapse were divided into seven cell subsets. The seven cell subsets were annotated into four cell types, including CMP, GMP, B cell and Pro-B_CELL_CD34 + (Figure 8F). In the diagnostic population, seven of the nine prognostic immune genes were found to be expressed in cell subsets. CD74 was found to be highly expressed in CMP, GMP, B cell, and Monocyte. IFI30 was highly expressed in GMP and Monocyte. However, almost none of the other five genes are expressed in cells. At the same time, seven of the nine prognostic immune genes were also found to be expressed in cell subsets in the relapse population. CD74 was found to be highly expressed in CMP, GMP, B cell and Monocyte. IFI30 was highly expressed in pro-B_cell_CD34 + and B cells. PTK2 was highly expressed in pro-B_cell_CD34 +. Almost none of the other four genes are expressed in cells.

### Pseudo-time analysis of cell subsets

To further explore the differentiation of different cell statuses, we simulated the movement trajectory of different cells in the diagnosed and relapsed AML population and observed the differentiation of cells. The cell track differentiation of the diagnosed patients is shown in Figure 7G–I; Supplementary Figure S3A. CMP cells here show two pathways of differentiation. One was CMP cells differentiated into GMP cells that subsequently gave rise to monocytes, and the other was Pro−B_cell_CD34+ and B cells. Cell track differentiation in patients with relapses is shown in Figures 8G–I; Supplementary Figure S4A. There were still two differentiation pathways shown here in CMP cells. One type of CMP was differentiated into GMP cells, and the other was differentiated into Pro−B_cell_CD34+ and B cells. The expression levels of the seven genes in different cell lines during differentiation in patients with diagnosed and relapsed AML patients were shown in Supplementary Figures S3B,C and Supplementary Figures S4B,C. We found that the expression levels of CD74 were high in all cell subsets. The expression level of PLXNB1 in the diagnosis group increased first and then gradually stabilized with the time of cell differentiation, but it was almost not expressed in the relapse group. The other five genes were almost unexpressed in all cell differentiation processes. Supplementary Figures S3D, 4D showed the plot of cell density versus pseudotime in patients with AML at diagnosis and relapse.

## 5 Discussion

Previous studies have shown that AML is a tumor with abnormal immune cell differentiation and is morphologically classified into eight subtypes from M0 to M7 (Bennett et al., 1976). The abnormal differentiation of hematopoietic stem cells exhibited by AML is inextricably linked to the expression of immune genes. The expression of tumor immune-related genes is the result of the interaction of immune cells, tumor stem cells, stromal cells and cytokines, which co-evolve and ultimately form the tumor microenvironment that supports the tumor, thus contributing to the development and progression of leukemia (Swartz et al., 2012). Although previous studies have obtained some biomarkers for the prognosis of AML (Jiang et al., 2022; Lu et al., 2022), no studies have explored the overall characteristics of AML-related TME and mutation. Therefore, our study not only explored the TME and mutation-related characteristics of AML but also analyzed the cell trajectory of immune-related prognostic genes obtained in single-cell sequencing chips to observe the expression of immune-related genes in different differentiated cells.

In our study, we divided the population into two groups based on the ssGSEA results using “hclust” method. Compared with the low immune cell infiltration cluster, higher stromal score, higher immune score and higher estimate score were shown in the high immune cell infiltration cluster. In addition to a large number of immune pathways, leukocyte transendothelial migration pathways and leukemia pathways were also enriched in patients with high immune cell infiltration clusters (Puig-Kröger et al., 2000). Since the migration of white blood cells from the blood into tissues is essential for immune surveillance and inflammation. This may mean that the high immune cell infiltration cluster may have more inflammatory responses and metastasis of AML cells.

High immune cell infiltration cluster associated with higher expression of HLA in all HLAs except HLA-J. It was reported that altered expression and function of HLA class I and class II molecules have long been characterized in solid tumors (Rovatti et al., 2020), while both HLA class I and class II antigens on the graft cell surface are strong transplant antigens (Salman et al., 2018). Besides, some studies have found a recurrence of transplantation in AML following genomic HLA loss. When we inject donor-derived T-cells into animals carrying a diagnosis of HLA II expression or relapse of HLA II deficiency, HLA II expression is restored and effective anti-leukemic response is re-established (Toffalori et al., 2019). Based on our results, the higher HLA expression in the high immune cell infiltration cluster than in the low immune cell infiltration cluster implies that the anti-leukemic response was superior in the high immune cell infiltration cluster.

Nine hub immune genes associated with AML prognosis were obtained by Cox analysis. All 9 genes were risk factors for AML prognosis in the forest plot (HR > 1). CD74 is found to be linked to LGALS3 in a protein network and associated with poor survival in AML (Ruvolo et al., 2019). Alterations in PLXNB1 exons are identified as a method of homozygous alteration in AML-associated isoforms (Risueño et al., 2014). There are no reports of its correlation with the prognosis of AML. THBS1 is found to be lowly expressed in AML patients. Patients with low THBS1 have a shorter survival time. So, THBS1 is considered as a possible prognostic target for the treatment of AML patients (Zhu et al., 2019). PTK2 is an adherent spot gene. Its overexpression contributes to poorer prognosis in leukemia in a cohort of AML patients. It can also distinguish subgroups of patients with poor prognosis among those with IR-AML cytogenetics and unfavorable FLT3/NPM1 combinations (Pallarès et al., 2018). UNC93B1 is a key regulator of Toll-like receptors (TLRs), pattern recognition receptors that sense invading pathogens and manage the innate immune response and deliver them from the endoplasmic reticulum to their respective endosomal signaling regions. Several types of TLRs are known to contribute to the inflammatory process after allogeneic hematopoietic stem cell transplantation (SCT). Thus, UNC93B1 may play an integral role in this process and influence the prognosis of leukemia (Uchino et al., 2021). PPBP is a proplatelet basic protein that belongs to the CXC chemokine family, High expression of PPBP predicts poor prognosis in adult AML patients (Tang et al., 2020). CXCL12 is produced by the BM microenvironment, binds to and activates the cognate receptor CXCR4 on leukemic cells, promotes transport and homing of leukemic cells in the BM microenvironment, and brings leukemic cells into close contact with stromal cells and the extracellular matrix, thereby constitutively producing growth-promoting and anti-apoptotic signals that ultimately lead to a poor prognosis (Roma-Rodrigues et al., 2019). GZMB is also considered to be a predictor of shorter OS in AML patients (Vadakekolathu et al., 2020). IFI30, a gene involved in antigen processing and HLA presentation, is observed to be transcriptionally downregulated in patients at the time of AML relapse (Sweeney and Vyas, 2019).

The areas under the ROC curve of the train set and the test set for ROC at 1, 3, 5 years were 0.807, 0.813, 0.815, and 0.731, 0.745, 0.830, respectively. The results showed that the model has strong predictive power for the prognosis of AML patients. We investigated the effect of risk score, gender, age, white blood cell count, FAB stage, hemoglobin, monocyte, race, and percentage of bone marrow blast on patient prognosis in AML patients by multivariate Cox regression analysis. Risk score and prognostic status were independent factors that influence the prognosis of leukemia (*p* < 0.001). Then a nomogram was designed that included risk scores and all clinical factors. It allowed us to predict the survival rate of any AML patient at 1, 3, and 5 years by using risk scores and all clinical factors from them. Interestingly, in some studies on biomarkers of leukemia prognosis, we found similar results to our article. Risk score was found to be an independent prognostic factor in these studies (Jiang et al., 2021b).

We constructed the TF hub gene regulatory network to explore the molecular mechanism of AML. A total of 10 transcription factors were found to play a role in the prognosis of AML immune gene expression. PPARD maintains leukemic stem cells through molecules involved in or regulating Wnt signaling, and is a valuable prognostic molecule (Gruszka et al., 2019). VDR functions as a regulator of stem cell homeostasis and leukemic transmission. The combination of VDR agonists and hypomethylating agents can promote leukemic stem cell depletion and reduce tumor burden (Paubelle et al., 2020). B cell lymphoma 6 (BCL6) is a transcriptional repressor and proto-oncogene that can maintain the survival and self-renewal of primary human acute myeloid leukemia cells (Kawabata et al., 2021). High-frequency eomes + T-bet low CD8 + T-cells predict poor clinical outcomes in AML, and targeting eomes may provide therapeutic benefits for AML (Jia et al., 2019). IRF4 expression is associated with the clinical phenotype and clinical hematological response of hydroxyurea in primary thrombocytosis, which may lead to the progression of AML (Huang et al., 2022). HDAC1 and KLF4 interact with each other to regulate the proliferation of human myeloid leukemia cells (Huang et al., 2014). IDH2/R140Q reduced 5 hmc modification and expression of some differentiation-inducing genes (ebf1 and SPIB). This is critical for the development and maintenance of AML stem cell-like cells (Ogawara et al., 2015). The complex interactions between TFs and hub genes have made great contributions to the development of AML.

AML patients were classified into high-risk and low-risk groups based on the median value of the risk score. Besides, we found that several other studies have also identified prognostic immune genes (Lu et al., 2022), but these studies have not systematically analyzed other characteristics of AML patients. In contrast to these studies, we not only constructed prognostic models of immune-related genes. We also comprehensively analyzed the characteristics of mutations, TME, HLA, and PPI in AML patients and identified the expression of these prognostic genes in single-cell sequencing analysis. Since we used immune-related genes to construct the prediction model, the GSEA results showed that we enriched a large number of immune-related pathways in the high-risk group. The expression of immune checkpoints was all higher in high-risk groups than in low-risk groups. Since high expression of immune checkpoints induces T-cell apoptosis or suppresses tumor T-cell responses, this leads to immune escape promoting the progression of AML (Christopher et al., 2018; Rovatti et al., 2020). Compared to the low-risk group, the high-risk group will have a worse prognosis. We found the worst prognosis for the high TMB and high-risk population and the best prognosis for the high TMB and low-risk population in the survival curves integrating TMB with risk profile. Based on the potential hypothesis that tumor mutations produce antigenic peptides, high TMB had been proposed as a prime candidate biomarker for the immunotherapeutic response, thereby enhancing immunogenicity (Chan et al., 2019). Therefore, high-risk with high TMB groups people should have the worst prognosis. Since there was no statistical difference in survival between the high TMB group people and the low TMB group people, the high TMB with the low risk group people might have the best prognosis. In Jiang’s study, we found similar results. The expression of ICI was higher in AML patients in the high-risk group (Jiang et al., 2021a). The results of immunotherapy response showed that the TIDE score and Dysfunction scores in the high-risk group were higher than those in the low-risk group, suggesting that patients in the low-risk group may be more sensitive to anti-PD-1 and anti-CTLA4 therapy. The immune microenvironment in high-risk patients is not conducive to ICI treatment because these patients do not benefit from these inhibitors.

Single-cell transcriptome analysis showed that patients with diagnosed AML and patients with relapsed AML had different cell types. Monocytes were the precursors of macrophages and dendritic cells and can influence the tumor microenvironment by inducing immune tolerance, angiogenesis and tumor cell proliferation. It can also induce an immune response that produces antitumor effectors and activates antigen presenting cells (Shen et al., 2021; Ugel et al., 2021). Monocyte subsets were present at the time of AML diagnosis but not at the time of relapse. It suggested that there may be impaired function of monocytes phagocytosis of tumor cells in the development of AML, resulting in immunosuppression and ultimately contributing to the relapse of AML. In addition, we found that CD74 was highly expressed during the development of all cells in both diagnosed and relapsed AML patients. CD74 is a type II transmembrane protein expressed on antigen-presenting cells and has been considered a viable therapeutic target for AML in children and adults (Le et al., 2021). Our results will provide further evidence for CD74 as a target for immunotherapy in AML.

Compared with previous studies, our study has some innovations. For example, compared to Lu’s study and Jiang’s study (Jiang et al., 2021a; Lu et al., 2022). We not only comprehensively analyzed the TME characteristics, human leukocyte antigen expression and mutation information of AML patients in this study but also used the external adult AML validation dataset to verify the robustness of our prediction model. More importantly, due to single-cell sequencing analysis, we analyzed the expression of prognostic immune genes in cell subpopulations and plotted the change curve of prognostic gene expression with cell trajectory differentiation. In addition, we have successfully correlated the expression of prognostic genes with cell differentiation trajectories and can provide some new insights for targeted therapy of AML patients.

We should acknowledge that our study still has some limitations. In further studies, larger-sample clinical cohorts are needed to validate the accuracy of the prognostic model and the nomogram. Due to the small number of cells detected by the chip selected in this study, some cell subsets could not be detected. Therefore, subsequent studies need to further expand the number of cell tests to present a more complete bone marrow immune microenvironment.

In conclusion, we identified different immune subtypes in AML patients and established a prognostic model with nine prognostic biomarkers to predict the prognosis of patients with different immune cell infiltration clusters. Meanwhile, we revealed the differentiation trajectory of bone marrow microenvironment cells and the expression of prognostic immune genes in AML patients. Our study provided a means to predict prognosis and survival in AML patients and may provide promising targets for immunotherapy.

## Data Availability

The original contributions presented in the study are publicly available. This data can be found https://github.com/zhengguowei-afk/code-and-input-data.git

## Supplementary material

SUPPLEMENTARY FIGURE S1 Comparison of clinical factors between high and low immune groups. (A) Age. (B) BM. (C) Hemoglobin. (D) Monocyte. (E) WB. (F) Calibration curve of train set. (G) Calibration curve of test set. (H) Distributions of risk scores of AML patients in the train set. (I) Distributions of survival status of AML patients in the train set. (J) Distributions of risk scores of AML patients in the test set. (K) Distributions of survival status of AML patients in the test set.

SUPPLEMENTARY FIGURE S2 Immune checkpoint inhibitor expression and TMB in high and low risk groups (A) Expression of HAVCR2 between high and low risk groups. (B) Expression of LAG3 between high and low risk groups. (C) Venn diagram of intersection of differential genes and immune genes. (D) Correlation between HAVCR2 and risk score. (E) Correlation between LAG3 and risk score. (F) Screening of genes by LASSO regression. (G) Differences in survival rates between high and low TMB populations. (H) Comparison of TMB between high-risk and low-risk groups. (I) The prognostic genes were screened by univariate Cox regression analysis.

SUPPLEMENTARY FIGURE S3 Partial pseudotime distribution of AML patients at diagnosis (A) Cell types on different differentiation trajectories. (B) Pseudotime distribution of seven genes between different cells. The horizontal axis is pseudo time, and the vertical axis is gene expression. (C) Pseudotime heatmap of 7 genes between different cells. The horizontal axis is pseudo time, and the vertical axis is gene expression. (D) Pseudotime distribution of different cell types. Pseudo time on the horizontal axis, cell density on the vertical axis.

SUPPLEMENTARY FIGURE S4 Partial pseudotime distribution of AML patients at relapse (A) Cell types on different differentiation trajectories. (B) Pseudotime distribution of seven genes between different cells. The horizontal axis is pseudo time, and the vertical axis is gene expression. (C) Pseudotime heatmap of 7 genes between different cells. The horizontal axis is pseudo time, and the vertical axis is gene expression. (D) Pseudotime distribution of different cell types. Pseudo time on the horizontal axis, cell density on the vertical axis.

SUPPLEMENTARY FIGURE S5 Univariate and multivariate Cox regression analysis of risk score and clinical factors (A) The expression levels of 9 AML prognostic genes were different between GTEx database and TCGA database. (B) Differential clustering heat map of the expression levels of 9 AML prognostic genes in GTEx database and TCGA database. (C) Univariate Cox regression analysis was performed for all clinical factors and risk scores. (D) Multivariate Cox regression analysis was performed for all clinical factors and risk scores.

SUPPLEMENTARY FIGURE S6 TIDE scores between high and low risk groups (A) Dysfunction scoring in high risk group and low risk group. (B) Exclusion scoring in high risk group and low risk group. (C) MSI scoring in high risk group and low risk group. (D) TIDE scoring in high risk group and low risk group.

## Abbreviations

AML, acute myelogenous leukemia; AUC, area under curve; PD-L1, programmed cell death 1 ligand 1; CIBERSORT, Cell type Identification by Estimating Relative Subpopulations of RNA Transcription; CTLA-4, cytotoxic T-lymphocyte antigen-4; ESTIMATE, Estimation of Stromal and Immune cells in Malignant Tumor tissues using Expression data; FPKM, Fragments per kilobase million; GSEA, Genome Set Enrichment Analysis; KEGG, Kyoto Encyclopedia of Genes and Genomes; LAG3, lymphocyte-activation gene 3; LASSO, least absolute shrinkage and selection operator; OS, overall survival; PD-1, programmed cell death 1; ROC, The time-dependent receiver operating characteristic; TCGA, The Cancer Genome Atlas; TIM-3, mucin domain-containing molecule-3; TMB, Tumor mutation burden. TIDE, Tumor Immune Dysfunction and Exclusion; TME, tumor microenvironment

The median age as well as the interquartile spacing is 57.50 (44.75, 67.00). The minimum value of age is 18, The maximum value of age is 88.Five variables were taken as covariates, including Gender, Age at Diagnosis in Days, Race, FAB Category, Ethnicity. “Gender” taken as categorical variable; “Race” taken as categorical variable; “Age at Diagnosis in Days”, expression of genes were taken as numerical variables; “FAB category” taken as categorical variable; “Ethnicity” were taken as categorical variable.
