# Supplementary figures and images for: Prognostic characteristics of immune subtypes associated with acute myeloid leukemia and their identification in cell subsets based on single-cell sequencing analysis
Source: Front Cell Dev Biol. 2022 Sep 23;10:990034. doi: 10.3389/fcell.2022.990034 (PMC9540204; doi:10.3389/fcell.2022.990034)

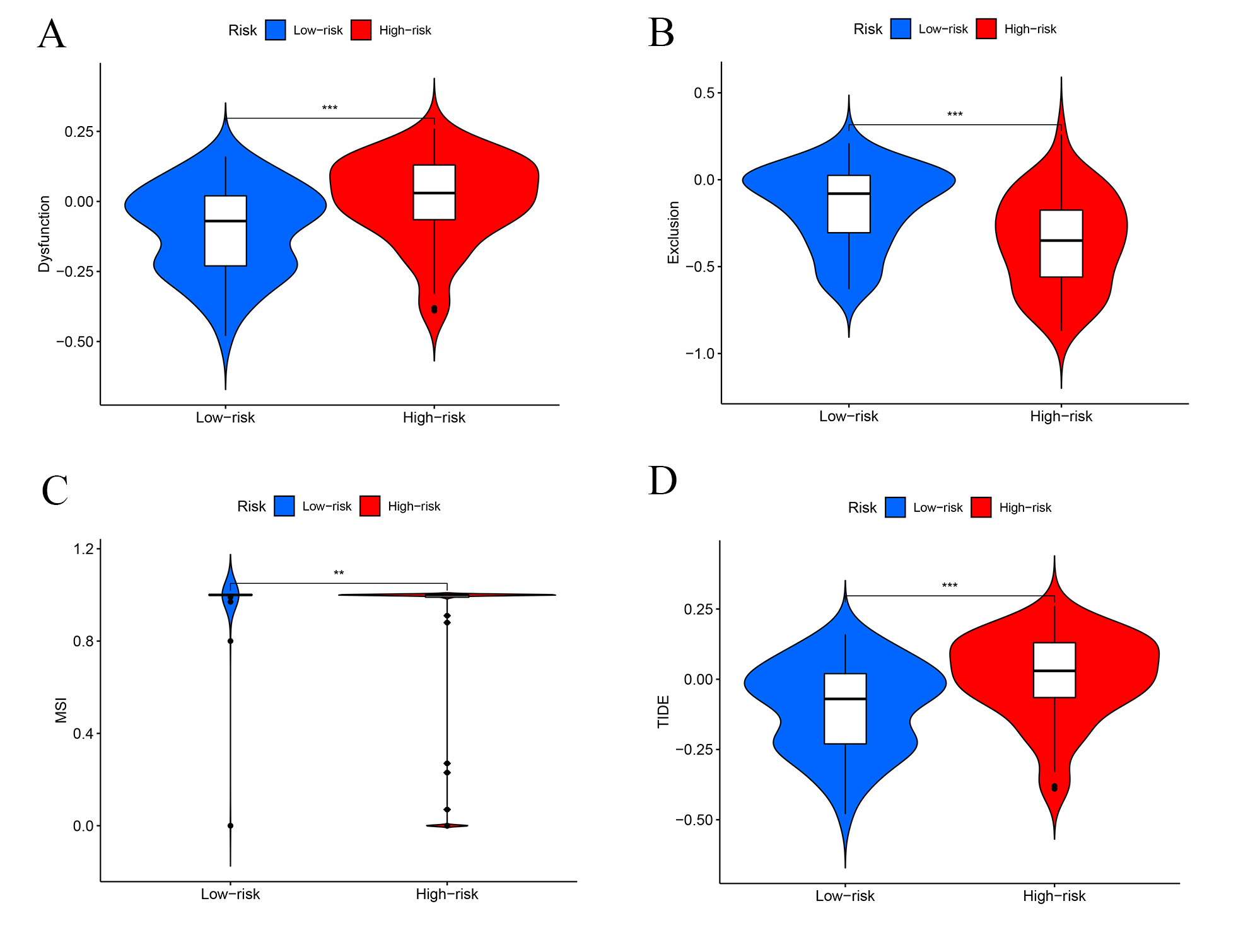

Supplement: Supplementary file 1 [file Image6.TIF]

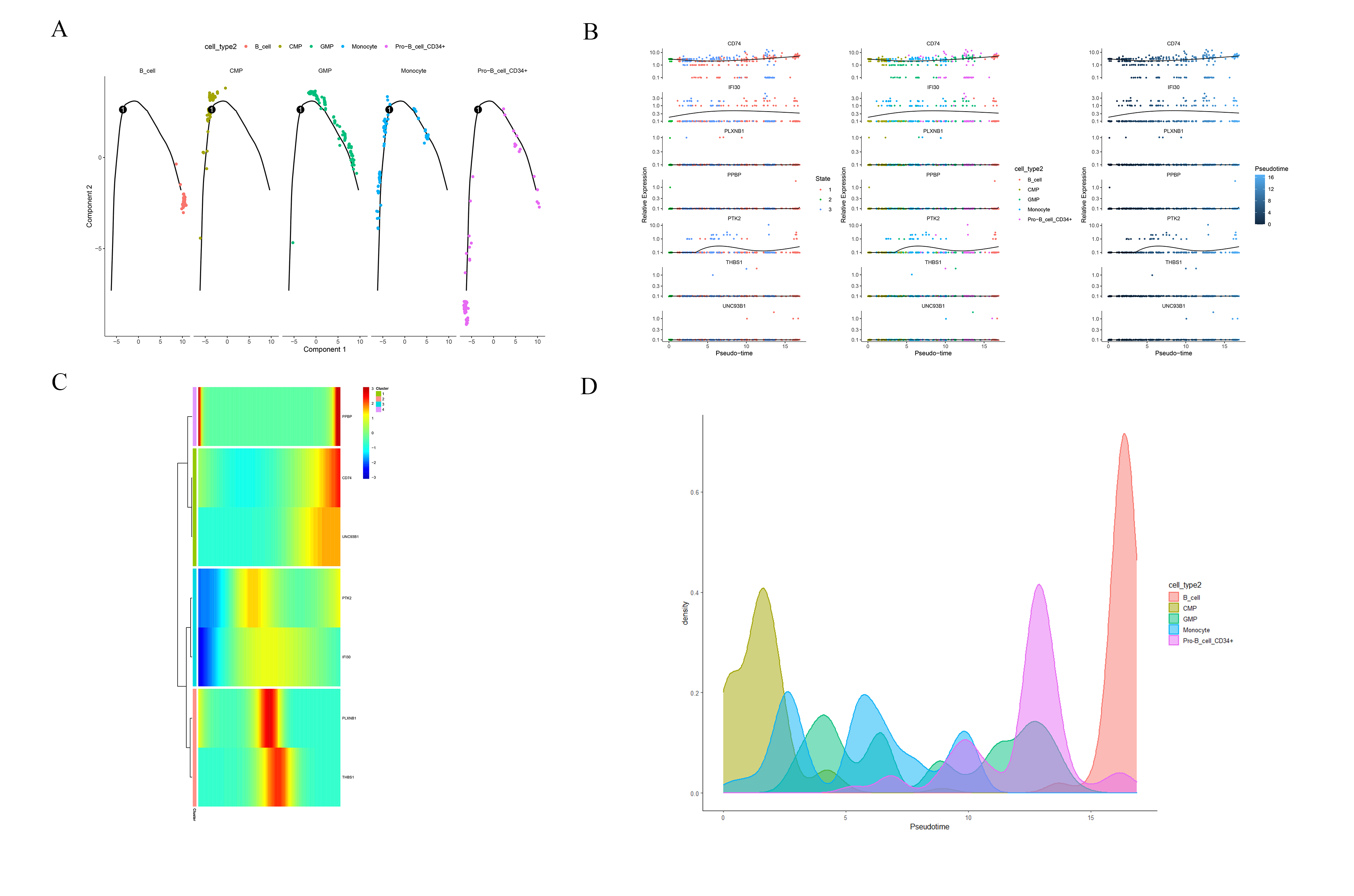

Supplement: Supplementary file 3 [file Image3.TIF]

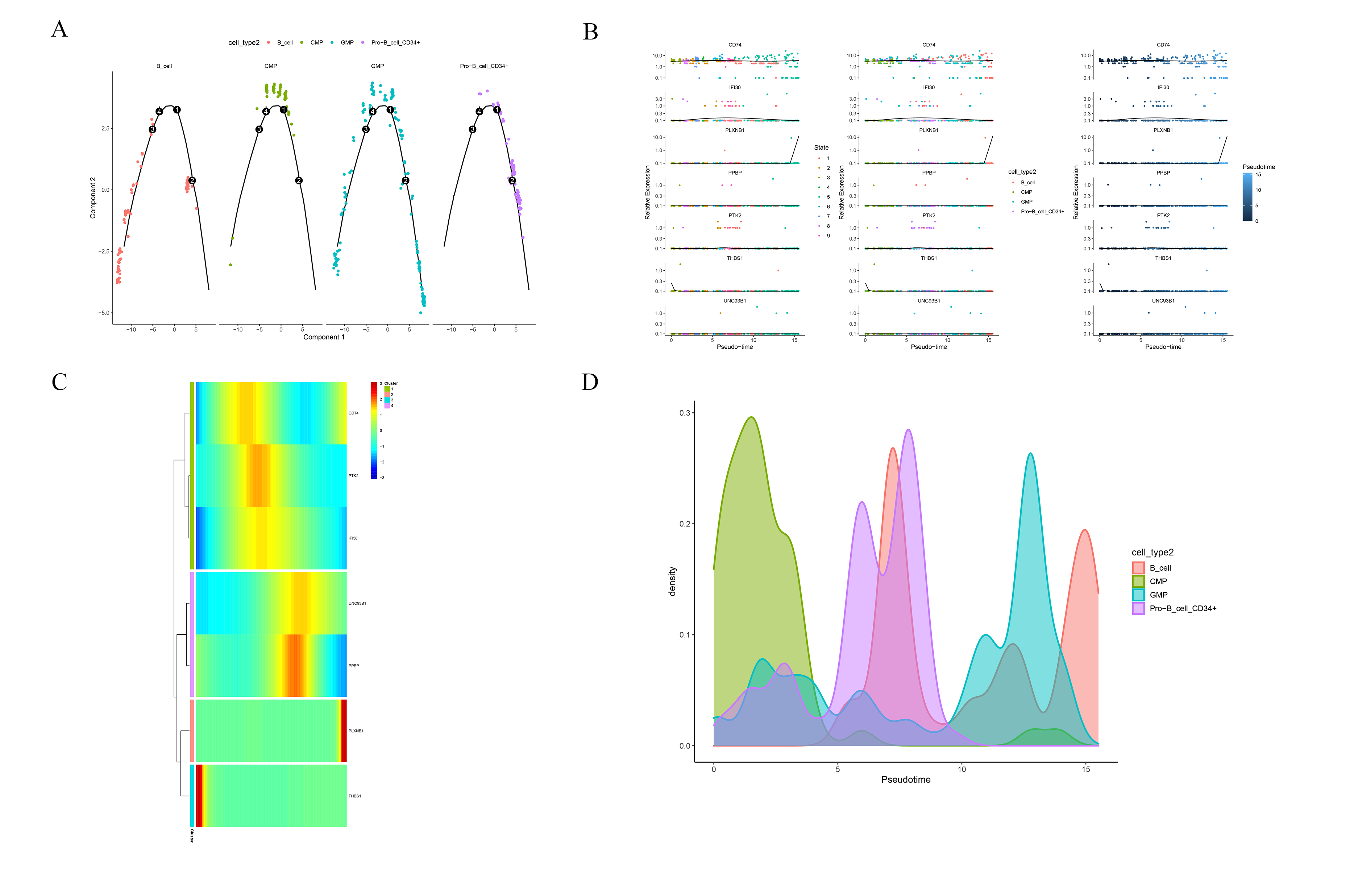

Supplement: Supplementary file 4 [file Image4.TIF]

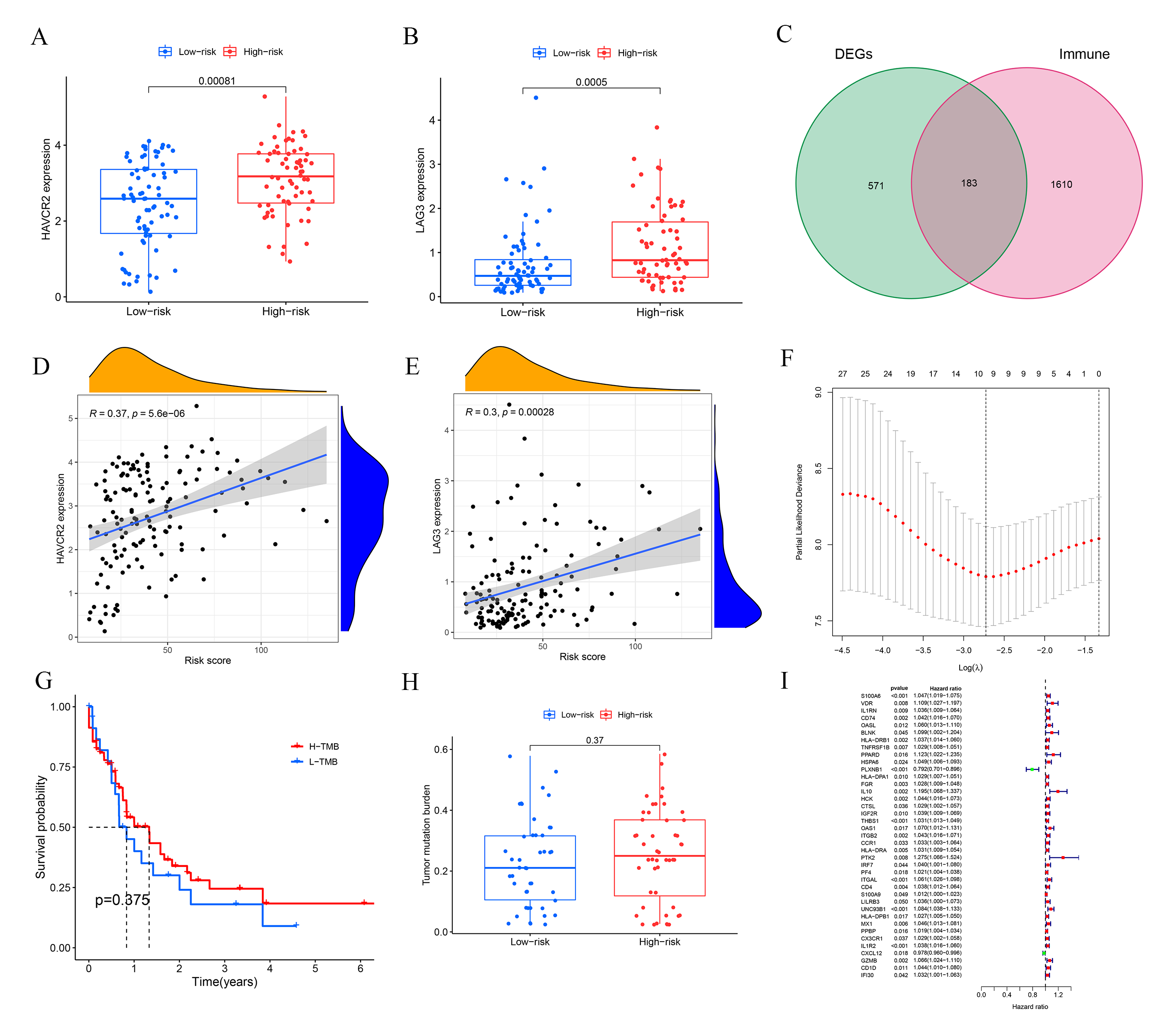

Supplement: Supplementary file 5 [file Image2.TIF]

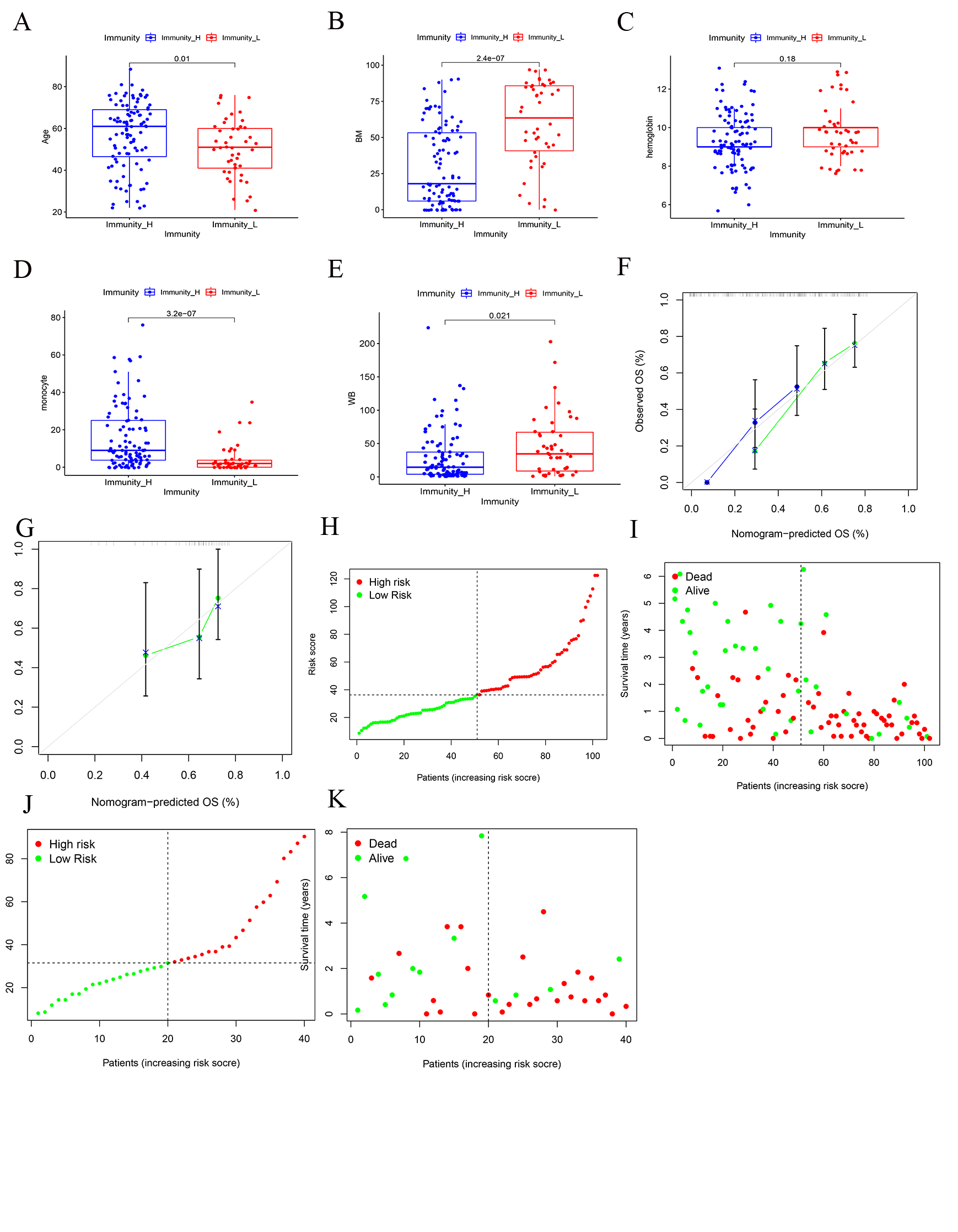

Supplement: Supplementary file 6 [file Image1.TIF]

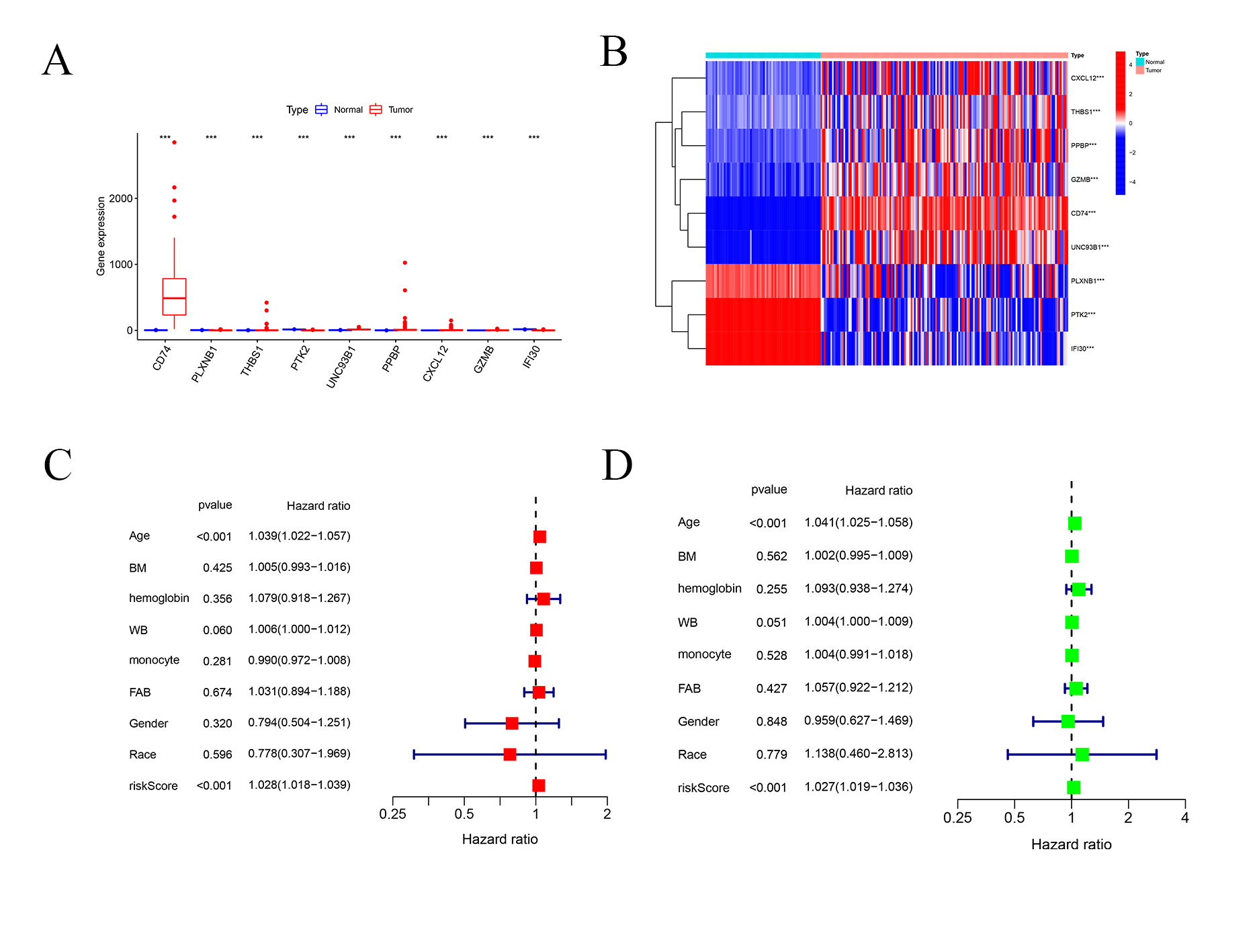

Supplement: Supplementary file 8 [file Image5.TIF]
