# Supplementary material for: Prognostic characteristics of immune subtypes associated with acute myeloid leukemia and their identification in cell subsets based on single-cell sequencing analysis
Source: Front Cell Dev Biol. 2022 Sep 23;10:990034. doi: 10.3389/fcell.2022.990034 (PMC9540204; doi:10.3389/fcell.2022.990034)
Supplement: Supplementary file 2 [file Table1.DOCX]

**Supplementary Table 1.** Baseline characteristics for 102 patients with AML in the training database (*n*=102)

|  | **characteristics** | **Cases (%)** |
| --- | --- | --- |
| Gender | | |
|  | Female | 45(43.7%) |
|  | Male | 57(55.3%) |
| Age | | |
|  | 10~ | 0 (0.0%) |
|  | 20~ | 10(9.7%) |
|  | 30~ | 12(11.7%) |
|  | 40~ | 15(14.6%) |
|  | 50~ | 18(17.5%) |
|  | 60~ | 27(26.2%) |
|  | 70~ | 17(16.5%) |
|  | 80~ | 3(2.9%) |
| Race | | |
|  | Asian | 1(1.0%) |
|  | Black or African American | 11(10.7%) |
|  | Not reported | 0(0.0%) |
|  | White | 90(87.4%) |
| FAB Category | | |
|  | M0 Undifferentiated | 9(8.7%) |
|  | M1 | 23(22.3%) |
|  | M2 | 25(24.3%) |
|  | M3 | 10(9.7%) |
|  | M4 | 20(19.4%) |
|  | M5 | 12(11.7%) |
|  | M6 | 2(1.9%) |
|  | M7 | 0(0.0%) |
|  | Not classified | 1(1.0%) |
| Ethnicity | | |
|  | Hispanic or Latino | 0(0.0%) |
|  | Not Hispanic or Latino | 101(98.1%) |
|  | Not reported | 1(1.0%) |
