# Supplementary material for: Prognostic characteristics of immune subtypes associated with acute myeloid leukemia and their identification in cell subsets based on single-cell sequencing analysis
Source: Front Cell Dev Biol. 2022 Sep 23;10:990034. doi: 10.3389/fcell.2022.990034 (PMC9540204; doi:10.3389/fcell.2022.990034)
Supplement: Supplementary file 7 [file Table2.DOCX]

**Supplementary Table 2.** Baseline characteristics for 40 patients with AML in the training database (*n*=40)

|  | **characteristics** | **Cases (%)** |
| --- | --- | --- |
| Gender | | |
|  | Female | 19(47.5%) |
|  | Male | 21(52.5%) |
| Age | | |
|  | 10~ | 0(0.0%) |
|  | 20~ | 3(7.5%) |
|  | 30~ | 5(12.5%) |
|  | 40~ | 4(10.0%) |
|  | 50~ | 10(25.0%) |
|  | 60~ | 10(25.0%) |
|  | 70~ | 8(20.0%) |
|  | 80~ | 0(0.0%) |
| Race | | |
|  | Asian | 0(0.0%) |
|  | Black or African American | 0(0.0%) |
|  | Not reported | 2(5.0%) |
|  | White | 38(95.0%) |
| FAB Category | | |
|  | M0 Undifferentiated | 5(12.5%) |
|  | M1 | 9(22.5%) |
|  | M2 | 9(22.5%) |
|  | M3 | 5(12.5%) |
|  | M4 | 8(20.0%) |
|  | M5 | 3(7.5%) |
|  | M6 | 0(0.0%) |
|  | M7 | 1(2.5%) |
|  | Not classified | 0(0.0%) |
| Ethnicity | | |
|  | Hispanic or Latino | 1(2.5%) |
|  | Not Hispanic or Latino | 37(92.5%) |
|  | Not reported | 2(5.0%) |
